# Supplementary figures and images for: Contribution of UbrA, a ubiquitin ligase essential for Arg/N-degron pathway, to peptidase gene expression in Aspergillus oryzae
Source: Appl Environ Microbiol. 2025 Sep 23;91(10):e00813-25. doi: 10.1128/aem.00813-25 (PMC12542663; doi:10.1128/aem.00813-25)

Fig. S1

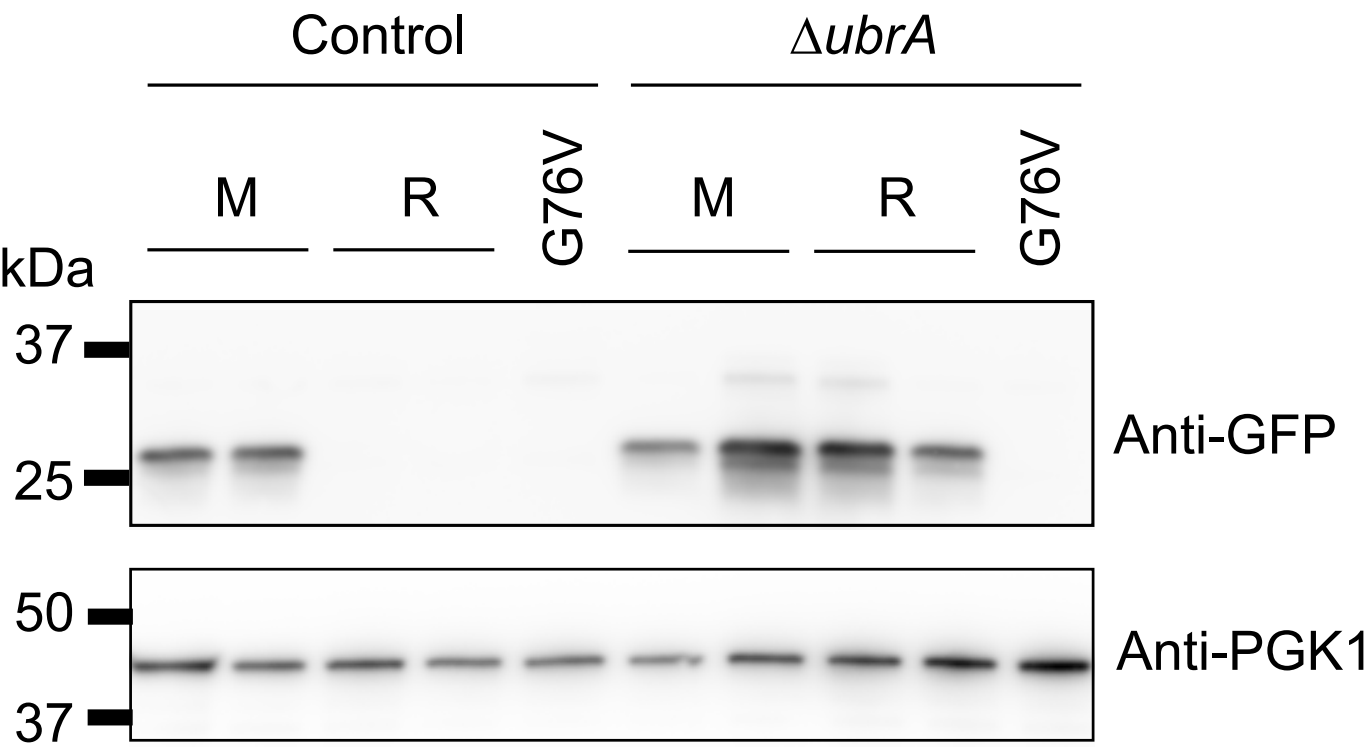

Supplement: Fig. S1 — Western blot analysis of Ub-M-GFP, Ub-R-GFP, and Ub-G76V-GFP expressed in the control and ∆ubrA strains. [file aem.00813-25-s0001.pdf]

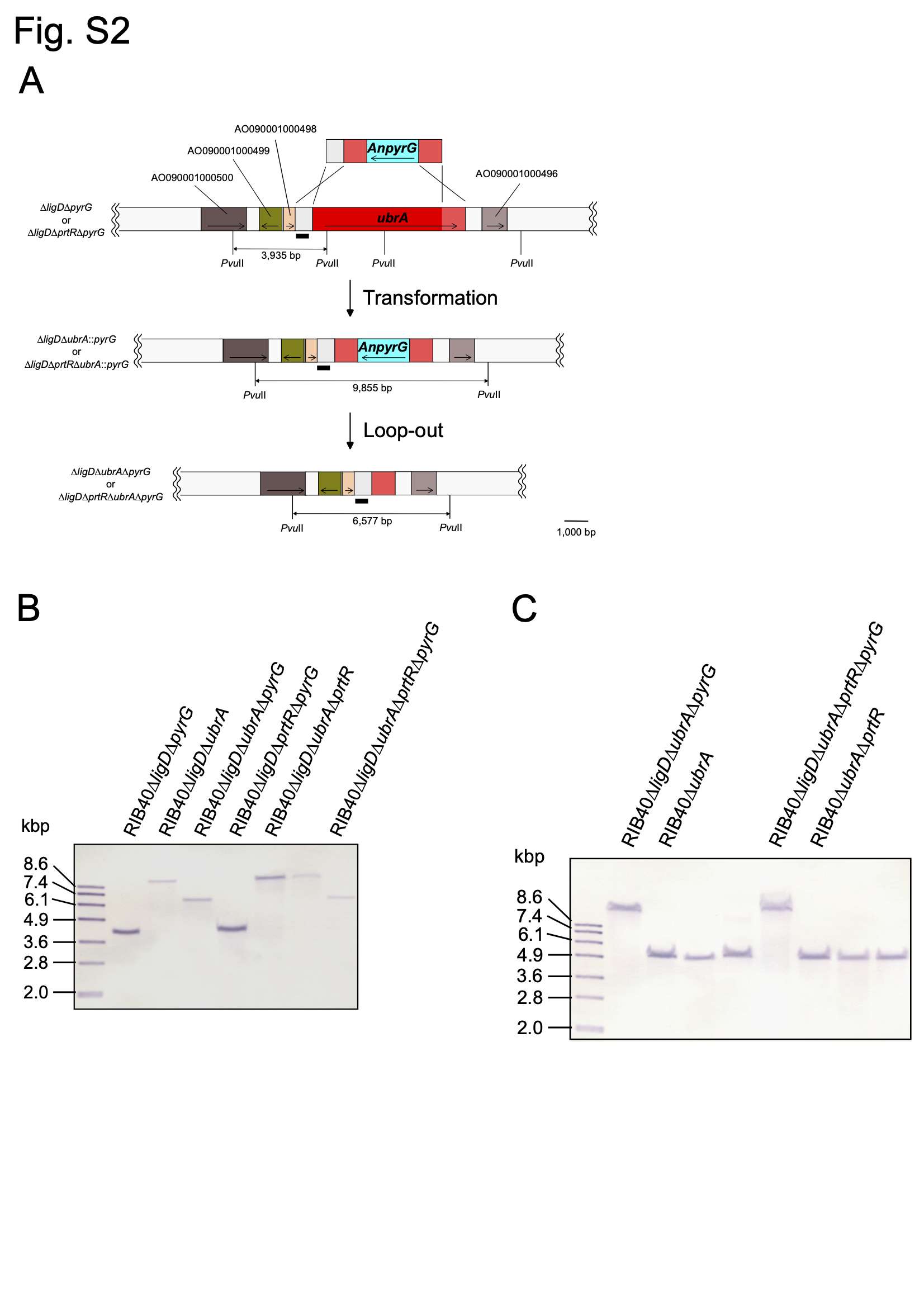

Supplement: Fig. S2 — Southern blot analysis of strains complemented with pyrG and ligD. [file aem.00813-25-s0002.tiff]

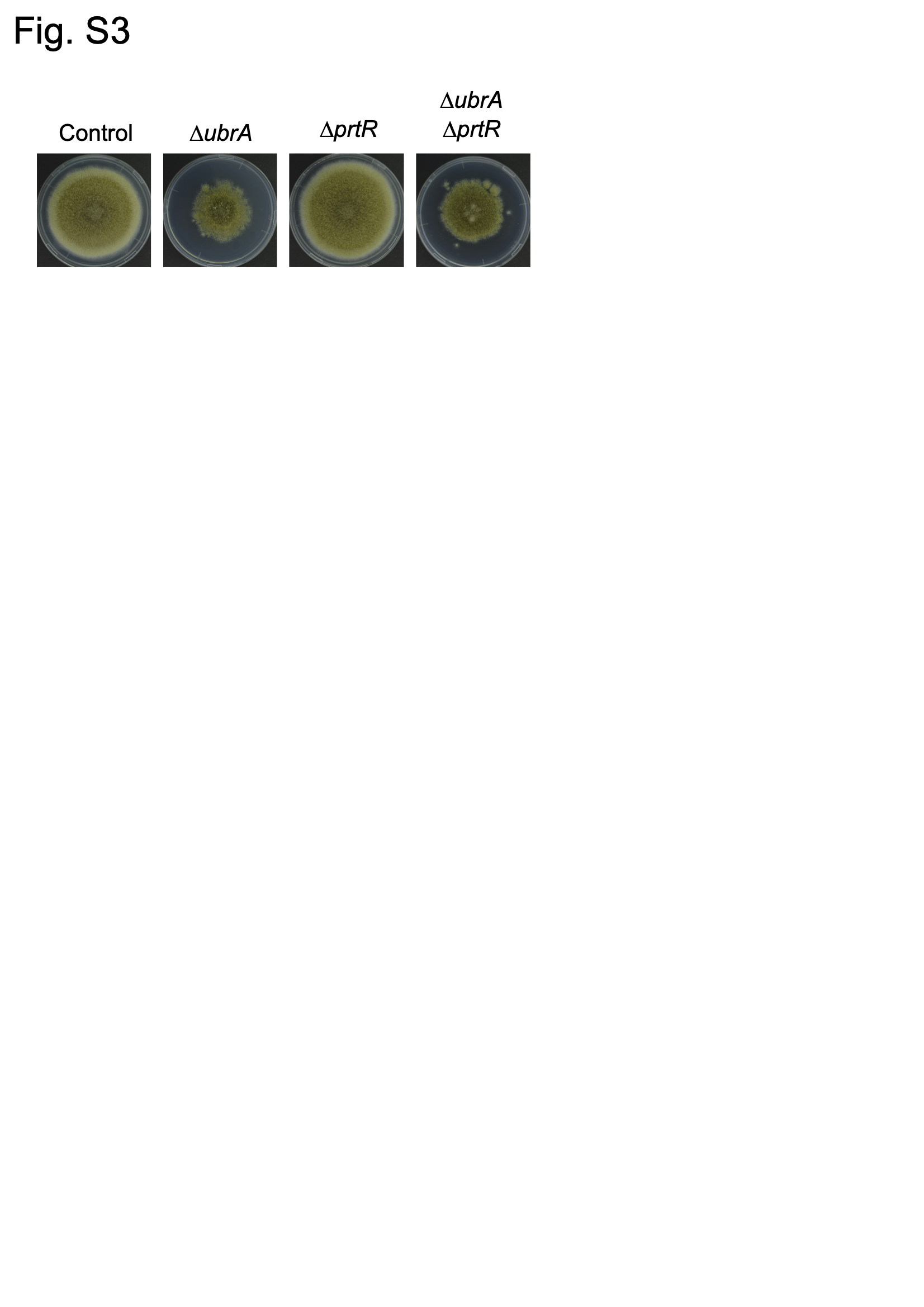

Supplement: Fig. S3 — Growth of ubrA disruption strains on CD agar medium. [file aem.00813-25-s0003.tiff]
