# Supplementary material for: Contribution of UbrA, a ubiquitin ligase essential for Arg/N-degron pathway, to peptidase gene expression in Aspergillus oryzae
Source: Appl Environ Microbiol. 2025 Sep 23;91(10):e00813-25. doi: 10.1128/aem.00813-25 (PMC12542663; doi:10.1128/aem.00813-25)
Supplement: Supplemental legends — Legends for Fig. S1 to S3. [file aem.00813-25-s0004.docx]

**Contribution of UbrA, a ubiquitin ligase essential for Arg/N-degron pathway, to peptidase genes expression in *Aspergillus oryzae***

**Running title: Involvement of UbrA in peptidase genes expression**

Waka Muromachi^1^, Mao Ohba^2^, Yasuaki Kawarasaki^2^, Youhei Yamagata^1^, Mizuki Tanaka^1#^

^1^ Department of Applied Biological Chemistry, Graduate School of Agricultural Science, Tokyo University of Agriculture and Technology, Fuchu, Tokyo, Japan.

^2^ Biomolecular Engineering Laboratory, School of Food and Nutritional Science, University of Shizuoka, Suruga-ku, Shizuoka, Japan.

^#^ Corresponding author:

Mizuki Tanaka, mizuki-tanaka@go.tuat.ac.jp

Key words: *Aspergillus oryzae*, N-degron pathway, proteolytic enzyme, transcription factor

**FIGURE LEGENDS**

Fig. S1. Western blot analysis of Ub-M-GFP, Ub-R-GFP, and Ub-G76V-GFP expressed in the control and *∆ubrA* strains. Approximately 2 × 10^7^ conidiospores of two independent transformants expressing Ub-M-GFP and Ub-R-GFP in both control and *∆ubrA* strains and one transformant expressing Ub-G76V-GFP in both control and *∆ubrA* strains were grown at 30 ºC for 20 h in liquid MM + 0.1% HIPOLYPEPTON N. After harvesting the mycelium, approximately 30 μg of extracted intracellular protein was subjected to western blot analysis using anti-GFP antibody. Phosphoglycerate kinase was detected as loading control by anti-PGK1 antibody.

Fig. S2. Generation of *ubrA* disruption strains to examine peptidase expression. (A) Construction of *ubrA* disruption. Thick black bar indicates probe for Southern blot analysis. (B) Southern blot analysis of *ubrA* disruption strains using genomic DNAs treated with *Pvu*II. The upstream region of *ubrA* was used as a probe. (C) Southern blot analysis of *ubrA* disruption strains complemented with *pyrG* and *ligD*. The *ptrA* marker in a *ligD* locus was replaced by a DNA fragment carrying the *pyrG* and *ligD* as described in Numazawa et al (2024) (33). The replacement at a *ligD* locus of *pyrG*-*ligD* fragment was confirmed using genomic DNA digested with *Pst*I and the *ligD* left probe (33).

Fig. S3. Growth of *ubrA* disruption strains on CD agar medium. Approximately 1 × 10^3^ conidiospores of each strain were grown on CD agar medium at 30 °C for 7 days.
