# Supplementary material for: Contribution of UbrA, a ubiquitin ligase essential for Arg/N-degron pathway, to peptidase gene expression in Aspergillus oryzae
Source: Appl Environ Microbiol. 2025 Sep 23;91(10):e00813-25. doi: 10.1128/aem.00813-25 (PMC12542663; doi:10.1128/aem.00813-25)
Supplement: Table S1 — A. oryzae strains used for this study. [file aem.00813-25-s0005.docx]

Table S1. *A. oryzae* strains used for this study

| Strain | Strain origin | Genotype | Reference |
| --- | --- | --- | --- |
| *∆ligD*::*ptrA* | NS4 | *niaD^-^*; *sC^-^*; *∆ligD*::*ptrA* | Mizutani et al. 2008 (55) |
| *∆ubrA* | *∆ligD::loxP pyrG^-^* | *niaD^-^*; *sC^-^*; *∆ligD*::*loxP*; *∆ubrA*::*pyrG* | Tanaka et al. 2021 (30) |
| RIB40*∆ligD∆pyrG* | RIB40 | *∆pyrG*; *∆ligD*::*ptrA* | Kobayashi et al. 2017 (33) |
| RIB40*∆ligD∆prtR∆pyrG* | RIB40*∆ligD∆pyrG* | *∆pyrG*; *∆ligD*::*ptrA*; *∆prtR*::*loxP* | Numazawa et al. 2024 (27) |
| Deletion-control | RIB40*∆ligD∆pyrG* | *∆pyrG*; *∆ligD*::*ligD-pyrG* | Kobayashi et al. 2017 (33) |
| RIB40*∆prtR* | RIB40*∆ligD∆prtR∆pyrG* | *∆pyrG*; *∆ligD*::*ligD-pyrG*; *∆prtR*::*loxP* | Numazawa et al. 2024 (27) |
| RIB40*∆ligD∆ubrA* | RIB40*∆ligD∆pyrG* | *∆ligD*::*ptrA*; *∆ubrA*::*pyrG* | This study |
| RIB40*∆ligD∆ubrA∆pyrG* | RIB40*∆ligD∆ubrA* | *∆pyrG*; *∆ligD*::*ptrA*; *∆ubrA* | This study |
| RIB40*∆ubrA* | RIB40*∆ligD∆ubrA∆pyrG* | *∆pyrG*; *∆ligD*::*ligD-pyrG*; *∆ubrA* | This study |
| RIB40*∆ligD∆ubrA∆prtR* | RIB40*∆ligD∆prtR∆pyrG* | *∆ligD*::*ptrA*; *∆prtR*::*loxP*; *∆ubrA*::*pyrG* | This study |
| RIB40*∆ligD∆ubrA∆prtR∆pyrG* | RIB40*∆ligD∆ubrA∆prtR* | *∆pyrG*; *∆ligD*::*ptrA*; *∆prtR*::*loxP*; *∆ubrA* | This study |
| RIB40*∆ubrA∆prtR* | RIB40*∆ligD∆ubrA∆prtR∆pyrG* | *∆pyrG*; *∆ligD*::*ligD-pyrG*; *∆prtR*::*loxP*; *∆ubrA* | This study |
| Ub-M-GFPsc | *∆ligD*::*ptrA* | *sC^-^*; *∆ligD*::*ptrA*; *niaD*::P*thiA*-Ub-M-GFP | This study |
| Ub-R-GFPsc | *∆ligD*::*ptrA* | *sC^-^*; *∆ligD*::*ptrA*; *niaD*::P*thiA*-Ub-R-GFP | This study |
| Ub-G76V-GFPsc | *∆ligD*::*ptrA* | *sC^-^*; *∆ligD*::*ptrA*; *niaD*::P*thiA*-Ub-G76V-GFP | This study |
| *∆ubrA* Ub-M-GFPsc | *∆ubrA* | *sC^-^*; *∆ligD*::*loxP*; *∆ubrA*::*pyrG*; *niaD*::P*thiA*-Ub-M-GFP | This study |
| *∆ubrA* Ub-R-GFPsc | *∆ubrA* | *sC^-^*; *∆ligD*::*loxP*; *∆ubrA*::*pyrG*; *niaD*::P*thiA*-Ub-R-GFP | This study |
| *∆ubrA* Ub-G76V-GFsc | *∆ubrA* | *sC^-^*; *∆ligD*::*loxP*; *∆ubrA*::*pyrG*; *niaD*::P*thiA*-Ub-G76V-GFP | This study |
| Ub-M-GFP | *∆ligD*::*ptrA* | *sC^-^*; *∆ligD*::*ptrA*; *niaD*::P*thiA*-Ub-M-GFP | This study |
| Ub-R-GFP | *∆ligD*::*ptrA* | *sC^-^*; *∆ligD*::*ptrA*; *niaD*::P*thiA*-Ub-R-GFP | This study |

Table S1. *A. oryzae* strains used for this study (continued)

| Strain | Strain origin | Genotype | Reference |
| --- | --- | --- | --- |
| Ub-A-GFP | *∆ligD*::*ptrA* | *sC^-^*; *∆ligD*::*ptrA*; *niaD*::P*thiA*-Ub-A-GFP | This study |
| Ub-D-GFP | *∆ligD*::*ptrA* | *sC^-^*; *∆ligD*::*ptrA*; *niaD*::P*thiA*-Ub-D-GFP | This study |
| Ub-E-GFP | *∆ligD*::*ptrA* | *sC^-^*; *∆ligD*::*ptrA*; *niaD*::P*thiA*-Ub-E-GFP | This study |
| Ub-C-GFP | *∆ligD*::*ptrA* | *sC^-^*; *∆ligD*::*ptrA*; *niaD*::P*thiA*-Ub-C-GFP | This study |
| Ub-P-GFP | *∆ligD*::*ptrA* | *sC^-^*; *∆ligD*::*ptrA*; *niaD*::P*thiA*-Ub-P-GFP | This study |
| Ub-S-GFP | *∆ligD*::*ptrA* | *sC^-^*; *∆ligD*::*ptrA*; *niaD*::P*thiA*-Ub-S-GFP | This study |
| Ub-W-GFP | *∆ligD*::*ptrA* | *sC^-^*; *∆ligD*::*ptrA*; *niaD*::P*thiA*-Ub-W-GFP | This study |
| Ub-F-GFP | *∆ligD*::*ptrA* | *sC^-^*; *∆ligD*::*ptrA*; *niaD*::P*thiA*-Ub-F-GFP | This study |
| Ub-I-GFP | *∆ligD*::*ptrA* | *sC^-^*; *∆ligD*::*ptrA*; *niaD*::P*thiA*-Ub-I-GFP | This study |
| Ub-L-GFP | *∆ligD*::*ptrA* | *sC^-^*; *∆ligD*::*ptrA*; *niaD*::P*thiA*-Ub-L-GFP | This study |
| Ub-G-GFP | *∆ligD*::*ptrA* | *sC^-^*; *∆ligD*::*ptrA*; *niaD*::P*thiA*-Ub-G-GFP | This study |
| Ub-H-GFP | *∆ligD*::*ptrA* | *sC^-^*; *∆ligD*::*ptrA*; *niaD*::P*thiA*-Ub-H-GFP | This study |
| Ub-N-GFP | *∆ligD*::*ptrA* | *sC^-^*; *∆ligD*::*ptrA*; *niaD*::P*thiA*-Ub-N-GFP | This study |
| Ub-V-GFP | *∆ligD*::*ptrA* | *sC^-^*; *∆ligD*::*ptrA*; *niaD*::P*thiA*-Ub-V-GFP | This study |
| Ub-K-GFP | *∆ligD*::*ptrA* | *sC^-^*; *∆ligD*::*ptrA*; *niaD*::P*thiA*-Ub-K-GFP | This study |
| Ub-Q-GFP | *∆ligD*::*ptrA* | *sC^-^*; *∆ligD*::*ptrA*; *niaD*::P*thiA*-Ub-Q-GFP | This study |
| Ub-T-GFP | *∆ligD*::*ptrA* | *sC^-^*; *∆ligD*::*ptrA*; *niaD*::P*thiA*-Ub-T-GFP | This study |
| Ub-Y-GFP | *∆ligD*::*ptrA* | *sC^-^*; *∆ligD*::*ptrA*; *niaD*::P*thiA*-Ub-Y-GFP | This study |
| *∆ubrA* Ub-M-GFP | *∆ubrA* | *sC^-^*; *∆ligD*::*loxP*; *∆ubrA*::*pyrG*; *niaD*::P*thiA*-Ub-M-GFP | This study |
| *∆ubrA* Ub-R-GFP | *∆ubrA* | *sC^-^*; *∆ligD*::*loxP*; *∆ubrA*::*pyrG*; *niaD*::P*thiA*-Ub-R-GFP | This study |

Table S1. *A. oryzae* strains used for this study (continued)

| Strain | Strain origin | Genotype | Reference |
| --- | --- | --- | --- |
| *∆ubrA* Ub-A-GFP | *∆ubrA* | *sC^-^*; *∆ligD*::*loxP*; *∆ubrA*::*pyrG*; *niaD*::P*thiA*-Ub-A-GFP | This study |
| *∆ubrA* Ub-D-GFP | *∆ubrA* | *sC^-^*; *∆ligD*::*loxP*; *∆ubrA*::*pyrG*; *niaD*::P*thiA*-Ub-D-GFP | This study |
| *∆ubrA* Ub-E-GFP | *∆ubrA* | *sC^-^*; *∆ligD*::*loxP*; *∆ubrA*::*pyrG*; *niaD*::P*thiA*-Ub-E-GFP | This study |
| *∆ubrA* Ub-C-GFP | *∆ubrA* | *sC^-^*; *∆ligD*::*loxP*; *∆ubrA*::*pyrG*; *niaD*::P*thiA*-Ub-C-GFP | This study |
| *∆ubrA* Ub-P-GFP | *∆ubrA* | *sC^-^*; *∆ligD*::*loxP*; *∆ubrA*::*pyrG*; *niaD*::P*thiA*-Ub-P-GFP | This study |
| *∆ubrA* Ub-S-GFP | *∆ubrA* | *sC^-^*; *∆ligD*::*loxP*; *∆ubrA*::*pyrG*; *niaD*::P*thiA*-Ub-S-GFP | This study |
| *∆ubrA* Ub-W-GFP | *∆ubrA* | *sC^-^*; *∆ligD*::*loxP*; *∆ubrA*::*pyrG*; *niaD*::P*thiA*-Ub-W-GFP | This study |
| *∆ubrA* Ub-F-GFP | *∆ubrA* | *sC^-^*; *∆ligD*::*loxP*; *∆ubrA*::*pyrG*; *niaD*::P*thiA*-Ub-F-GFP | This study |
| *∆ubrA* Ub-I-GFP | *∆ubrA* | *sC^-^*; *∆ligD*::*loxP*; *∆ubrA*::*pyrG*; *niaD*::P*thiA*-Ub-I-GFP | This study |
| *∆ubrA* Ub-L-GFP | *∆ubrA* | *sC^-^*; *∆ligD*::*loxP*; *∆ubrA*::*pyrG*; *niaD*::P*thiA*-Ub-L-GFP | This study |
| *∆ubrA* Ub-G-GFP | *∆ubrA* | *sC^-^*; *∆ligD*::*loxP*; *∆ubrA*::*pyrG*; *niaD*::P*thiA*-Ub-G-GFP | This study |
| *∆ubrA* Ub-H-GFP | *∆ubrA* | *sC^-^*; *∆ligD*::*loxP*; *∆ubrA*::*pyrG*; *niaD*::P*thiA*-Ub-H-GFP | This study |
| *∆ubrA* Ub-N-GFP | *∆ubrA* | *sC^-^*; *∆ligD*::*loxP*; *∆ubrA*::*pyrG*; *niaD*::P*thiA*-Ub-N-GFP | This study |
| *∆ubrA* Ub-V-GFP | *∆ubrA* | *sC^-^*; *∆ligD*::*loxP*; *∆ubrA*::*pyrG*; *niaD*::P*thiA*-Ub-V-GFP | This study |
| *∆ubrA* Ub-K-GFP | *∆ubrA* | *sC^-^*; *∆ligD*::*loxP*; *∆ubrA*::*pyrG*; *niaD*::P*thiA*-Ub-K-GFP | This study |
| *∆ubrA* Ub-Q-GFP | *∆ubrA* | *sC^-^*; *∆ligD*::*loxP*; *∆ubrA*::*pyrG*; *niaD*::P*thiA*-Ub-Q-GFP | This study |
| *∆ubrA* Ub-T-GFP | *∆ubrA* | *sC^-^*; *∆ligD*::*loxP*; *∆ubrA*::*pyrG*; *niaD*::P*thiA*-Ub-T-GFP | This study |
| *∆ubrA* Ub-Y-GFP | *∆ubrA* | *sC^-^*; *∆ligD*::*loxP*; *∆ubrA*::*pyrG*; *niaD*::P*thiA*-Ub-Y-GFP | This study |
