# Supplementary material for: Contribution of UbrA, a ubiquitin ligase essential for Arg/N-degron pathway, to peptidase gene expression in Aspergillus oryzae
Source: Appl Environ Microbiol. 2025 Sep 23;91(10):e00813-25. doi: 10.1128/aem.00813-25 (PMC12542663; doi:10.1128/aem.00813-25)
Supplement: Table S2 — Primers used in this study. [file aem.00813-25-s0006.docx]

Table S2 Primers used in this study

| Primer name | Nucleotide sequence (5' -3') |
| --- | --- |
| Ub-GFPsen MTS382 | GACAAGCTTGCGGCCGCCATGCAGATCTTCGTGAAGAC |
| Ub-GFPanti MTS383 | AGTCACGTGGCGGCCGCTTTACTTGTACAGC |
| niaDantiIFsC MTS471 | CGACTCTAGAGGATCGATATGTACTGGTCCGTTCTCG |
| TagdA-niaDanti MTT576 | ACCGAGCGTACACCACCGCACCAGCTCTTCTTG |
| niaD5-TagdAanti MTT577 | GAAGAGCTGGTGCGGTGGTGTACGCTCGGTAAAGTTG |
| niaD3-PthiAsen MTT578 | GAAAACAAGCTCCACCGCAATTGATTACGGGATCCCATTGG |
| PthiA-niaD3sen MTT579 | CCCGTAATCAATTGCGGTGGAGCTTGTTTTCGAC |
| niaD3anti MTT580 | CCTACGCCTGCTTAAACGATTCC |
| Ub-A-GFPsen2 MTT621 | AGAGGTGGGGCCGGGAAGCTTGGTCGACAG |
| Ub-A-GFPanti2 MTT622 | AAGCTTCCCGGCCCCACCTCTGAGACGGAGTAC |
| Ub-D-GFPsen2 MTT623 | AGAGGTGGGGATGGGAAGCTTGGTCGACAG |
| Ub-D-GFPanti2 MTT624 | AAGCTTCCCATCCCCACCTCTGAGACGGAGTAC |
| Ub-E-GFPsen3 MTT633 | AGAGGTGGGGAGGGGAAGCTTGGTCG |
| Ub-E-GFPanti3 MTT634 | AAGCTTCCCCTCCCCACCTCTGAGACG |
| Ub-F-GFPsen2 MTT635 | AGAGGTGGGTTCGGGAAGCTTGGTCGACAG |
| Ub-F-GFPanti2 MTT636 | AAGCTTCCCGAACCCACCTCTGAGACG |
| Ub-G-GFPsen2 MTT637 | AGAGGTGGGGGCGGGAAGCTTGGTCGACAG |
| Ub-G-GFPanti2 MTT638 | AAGCTTCCCGCCCCCACCTCTGAGACGGAGTAC |
| Ub-H-GFPsen2 MTT639 | AGAGGTGGGCATGGGAAGCTTGGTCGACAG |
| Ub-H-GFPanti2 MTT640 | AAGCTTCCCATGCCCACCTCTGAGACGGAGTAC |
| Ub-I-GFPsen2 MTT641 | AGAGGTGGGATCGGGAAGCTTGGTC |
| Ub-I-GFPanti2 MTT642 | AAGCTTCCCGATCCCACCTCTGAGAC |
| Ub-K-GFPsen2 MTT643 | AGAGGTGGGAAGGGGAAGCTTGG |
| Ub-K-GFPanti2 MTT644 | AAGCTTCCCCTTCCCACCTCTGAG |
| Ub-L-GFPsen2 MTT645 | AGAGGTGGGCTCGGGAAGCTTGGTCGACAG |
| Ub-L-GFPanti2 MTT646 | AAGCTTCCCGAGCCCACCTCTGAGAC |
| Ub-N-GFPsen2 MTT647 | AGAGGTGGGAACGGGAAGCTTGGTCGAC |
| Ub-N-GFPanti2 MTT648 | AAGCTTCCCGTTCCCACCTCTGAGACGG |

Table S2 Primers used in this study (continued)

| Primer name | Nucleotide sequence (5' -3') |
| --- | --- |
| Ub-P-GFPsen2 MTT649 | AGAGGTGGGCCTGGGAAGCTTGGTCGACAG |
| Ub-P-GFPanti2 MTT650 | AAGCTTCCCAGGCCCACCTCTGAGACGGAGTAC |
| Ub-Q-GFPsen2 MTT651 | AGAGGTGGGCAGGGGAAGCTTGGTCG |
| Ub-Q-GFPanti2 MTT652 | AAGCTTCCCCTGCCCACCTCTGAGACG |
| Ub-C-GFPsen2 MTT653 | AGAGGTGGGTGCGGGAAGCTTGGTCGACAG |
| Ub-C-GFPanti2 MTT654 | AAGCTTCCCGCACCCACCTCTGAGACGGAGTAC |
| Ub-S-GFPsen2 MTT655 | AGAGGTGGGTCCGGGAAGCTTGGTCGACAG |
| Ub-S-GFPanti2 MTT656 | AAGCTTCCCGGACCCACCTCTGAGACGGAGTAC |
| Ub-T-GFPsen2 MTT657 | AGAGGTGGGACCGGGAAGCTTGGTCGAC |
| Ub-T-GFPanti2 MTT658 | AAGCTTCCCGGTCCCACCTCTGAGACGG |
| Ub-V-GFPsen2 MTT659 | AGAGGTGGGGTCGGGAAGCTTGGTCGAC |
| Ub-V-GFPanti2 MTT660 | AAGCTTCCCGACCCCACCTCTGAGACG |
| Ub-W-GFPsen2 MTT661 | AGAGGTGGGTGGGGGAAGCTTGGTCG |
| Ub-W-GFPanti2 MTT662 | AAGCTTCCCCCACCCACCTCTGAGACG |
| Ub-Y-GFPsen2 MTT663 | AGAGGTGGGTACGGGAAGCTTGGTCGACAG |
| Ub-Y-GFPanti2 MTT664 | AAGCTTCCCGTACCCACCTCTGAGACGGAGTAC |
| ubrAup_F1 | CGGTACCCGGGGATCGCAGTAATTGATGGAGCCATTCTGG |
| ubrAup_R2 | TCGTCTCTAGTCTGAGAAGACGAGACGC |
| ubrAdown_F5 | ATGGAGATCTCGACCTTGGATATAGAGCTTTCGGATGAGCG |
| ubrAdown_R6 | CGACTCTAGAGGATCCCTAGATAGTTTCCCAGCCTCCATTG |
| ubrAdown_loopF3 | TCAGACTAGAGACGATTGGATATAGAGCTTTCGGATGAGCG |
| ubrAdown_loopR4 | AGGCGCTGCAGCAGGCCTAGATAGTTTCCCAGCCTCCATTG |
| AnpyrGsen | GGTCGAGATCTCCATCCCCGCAAACTAC |
| AnpyrGantiPstI | GTTATATGAGCCCAAGGCGCTGCAGCAGG |
| ubrAprobeF15 | GGAAGGTTGTCGGTCCCATG |
| ubrAprobeR16 | GGGATCGATGTCACTGTGGG |
| actA-RT-F | TCATGAAGTGTGATGTTGATGTCC |
| actA-RT-R | GGCAAGGGCGGTGATTTC |

Table S2 Primers used in this study (continued)

| Primer name | Nucleotide sequence (5' -3') |
| --- | --- |
| pepO-RT-F | CTCCTATGACTTCGGCTTCATC |
| pepO-RT-R | GCCCTTTCCGACAGAGTAACC |
| deuA-RT-F | CGCCAACTGCGACATCTAC |
| deuA-RT-R | CATAGCCGTAGCCCAAGTCC |
| deuB-RT-F | CGCCAACTGCGACATCTAC |
| deuB-RT-R | CATAGCCGTAGCCCAAGTCC |
| pipA-RT-F | ATCTCCATCCGCAACAGC |
| pipA-RT-R | AGCAGGCGTGACCAACAAG |
| np1-RT-F | CATCCGTGCTTACCCATTCTC |
| np1-RT-R | CCTTGAACTCGGGCTTTG |
| np3-RT-F | ACGGAATGGGAATGTGGGTTT |
| np3-RT-R | CAGGGATGTGTAGTTCAGTGGGT |
| aorA-RT-F | TTCGGGTGGTGGCTTCAG |
| aorA-RT-R | ATGGATAGGGAGGGTTGTGG |
| aorB-RT-F | CGAAGAACAAGCAACGACCAG |
| aorB-RT-R | CGCTTTCGTAGTAGGGATAGG |
| ocpA-RT-F | GAAGCAGGTAGTCAGAAAGGTG |
| ocpA-RT-R | CGATGAGTTGTCTGTAGGTGTTG |
| ocpB-RT-F | GAGCAGAACAACACATTTTACG |
| ocpB-RT-R | CGTTTTCGGTGGTATTGGC |
| ocpO-RT-F | CTACTTCAACCGCACTGACG |
| ocpO-RT-R | GTTCGTCCGCTCAATCACAC |
| prtR-RT-F | GTTCTCAATGCTCGTTCGGA |
| prtR-RT-R | GAAATGAGTGTGGCATGACCTC |
| flbC-RT-F MTS211 | CCCTCCAACACTCAGAAGAAG |
| flbC-RT-R MTS212 | CCTCTACATCACAGGCGAAAG |
| areA-RT-F | GGTAACCCTCAGTCAACCGACCTG |
| areA-RT-R | GTCGGAAATCTTGACGGCACTG |

Table S2 Primers used in this study (continued)

| Primer name | Nucleotide sequence (5' -3') |
| --- | --- |
| creA-RT-F | CTTCCCCGTCCATACAAGTGTCC |
| creA-RT-R | CGTATGTGTGCGAATATGTCTGGTC |
| amdX-RT-F | GCGAGGGACTGACATCGTCA |
| amdX-RT-R | CCCCAAGGTCTGCCACTGAA |
| xprG-RT1-F | TACTCGCGGACAGCAGAAAC |
| xprG-RF1-R | TCGGGCAGATCATTCAAAC |
| alpA-RT-Fw | CTTTCTGCCTGGATTGGCTC |
| alpA-RT-Rv | CAACTCCTTGATGCGCTTGG |
| pacC-RT-F | ACCTCTCATATCCGAGTACATGTGC |
| pacC-RT-R | TCAGGTGACCGAACCAGAACC |
| pacA1-RT-F19 | CGGCAACTGTTCAGCTGGTC |
| pacA1-RT-R20 | TAGGGGAAGGGGCTCGTGTA |
| sltB-RT-F30 | CCACTACCAGAGCAGCTTCC |
| sltB-RT-R31 | TATGGGGTGGAGACCTCTCG |
| potA-RT-F MTS52 | CTTACTCCTGCCGCTTACG |
| potA-RT-R MTS53 | GGTTCAGATGGCGGTACAC |
| potB-RT-F MTS66 | CCACGGTGATCCAACACTAC |
| potB-RT-R MTS67 | AGACGGAGATGGGCGAATAC |
| potC-RT-F MTS76 | CGACGGAGTTTCCTTGGTG |
| potC-RT-R MTS77 | GAGTAGGCGATACCGTAGGC |
| dppB-RT-F | CCTGTTTCTGACTGGCGTTTC |
| dppB-RT-R | CTCGTAGCCCTCCTCATTGGTC |
| dppE-RT-F | CAAACGGCACAGCATACAACGAAG |
| dppE-RT-R | CGGCATTGAATCGGGTGGTG |
| dppF-RT-F | CATGAGCTTGGTCGCCGATTC |
| dppF-RT-R | GCTCCTCAGTGCTCAACAGAG |
| tppA-RT-F | ATTGGCTTGATGGGTCTGCG |
| tppA-RT-R | AGTCTCGGTGCCGTCGTTAG |

Table S2 Primers used in this study (continued)

| Primer name | Nucleotide sequence (5' -3') |
| --- | --- |
| tppB-RT-F | ATGACCAACGACGGCACCAAC |
| tppB-RT-R | AGAAACCGCCCGAGGAGAAG |
| tppC-RT-F | CCAAATGGGAAGGGCTCTACAAC |
| tppC-RT-R | ACAATACCAGCAAACACAGGCG |
